# Preliminary investigation of equine veterinary hospital staff attitudes towards pain assessment in a single centre

**DOI:** 10.1002/vetr.6122

**Published:** 2025-12-04

**Authors:** Olivia Curry, Alice Everett, Gemma Pearson, Cathy Dwyer, Juliet Duncan

**Affiliations:** ^1^ The University of Edinburgh Royal Dick School of Veterinary Studies Edinburgh UK; ^2^ The Horse Trust Princes Risborough UK; ^3^ SRUC School of Veterinary Medicine and Biosciences Edinburgh UK; ^4^ Present address: Links Veterinary Group Haddington UK

**Keywords:** clinical practice, horses, pain

## Abstract

**Background:**

Despite the availability of several equine pain assessment tools, their use in equine veterinary practice appears limited compared to small animal practice. This study explores potential barriers to equine pain assessment, as reported by staff at a single UK equine teaching hospital.

**Methods:**

Nine hospital staff members were interviewed using semi‐structured interviews. Key themes were identified through reflexive thematic analysis.

**Results:**

Participants acknowledged the importance of pain assessment, yet highlighted limitations in current methods and their inconsistent use. Key challenges included limited observer confidence, subjective interpretations and discrepancies between staff and owner perceptions. Variability in horse temperament and pain presentation further complicated the assessment. Staff expressed a desire for improvements in pain assessment tools and clearer protocols.

**Limitations:**

The study was limited by its single‐hospital design, short interview duration and small sample size.

**Conclusion:**

The study highlights the complexity of equine pain assessment in clinical practice, including tool limitations, knowledge gaps and contextual barriers. Despite valuing pain assessment, staff reported difficulties applying currently available methods. Findings suggest a need for improved tools, training and institutional support.

## INTRODUCTION

The accurate assessment of pain and its treatment is a crucial aspect of equine veterinary medicine and welfare because, if left untreated, the experience of pain can cause significant distress and suffering.^1^, ^2^ However, despite advancements in pain assessment methods, their routine use in equine practice is limited.^3^, ^4^ The discrepancy between the availability of tools and their implementation suggests certain factors, such as job roles and responsibilities,^5^, ^6^, ^7^ training and education,^8^, ^9^ previous experiences^10^ and years since graduation,^11^ may influence how veterinary professionals approach pain assessment in horses. While these factors may not directly act as barriers, they may contribute to the variability in the consistent adoption of pain assessment tools.

The absence of standardised pain assessment practices in equine medicine is particularly evident compared to small animal medicine. For instance, the World Small Animal Veterinary Association (WSAVA) guidelines emphasise the importance of pain assessment, stating ‘Pain should be assessed using validated pain scales/scoring systems/tools’.^12^ Similarly, the American Animal Hospital Association (AAHA) and American Association of Feline Practitioners (AAFP) Pain Management Guidelines recommend the use of pain‐scoring tools for both acute and chronic pain. They further emphasise that ‘pain assessment should be a routine component of every physical examination, and a pain score is considered the ‘fourth vital sign’, after temperature, pulse and respiration’.^13^

By contrast, equine medicine lacks an equivalent ‘gold standard’ pain assessment method,^14^ as noted in the British Equine Veterinary Association (BEVA) primary care clinical guidelines, which call for further research.^15^ Unlike small animal practice, where heightened awareness of pain management^16^ has led to the more routine use of validated and widely adopted pain assessment tools, equine medicine has seen less widespread adoption. For example, a 2023 study^17^ reported that 55.9% of veterinary professionals in the UK treating dogs used the Glasgow Composite Measure Pain Scale (CMPS), an increase from 17% of small animal veterinary surgeons in 2013^18^ and 8.1% of veterinary nurses in 2004 who used pain scales.^19^ Globally, the adoption of pain assessment tools in small practices is also increasing. In the United States, 47.6% of companion animal veterinary surgeons and technicians reported routinely performing pain scoring. Similarly, in Spain, 82% of small animal veterinary surgeons assessed pain, although only 28% used formal, validated pain scales. Of those using scales, 94% used the CMPS, and 85% of those not using a pain scale expressed interest in incorporating one into their practice.^9^

However, while small animal medicine has made significant progress in both the adoption of pain assessment tools^20^, ^21^ and research into the barriers to their implementation,^8^, ^9^, ^22^ similar studies are notably absent in equine practice. While this scarcity of research does not necessarily imply an absence of implementation, it highlights a critical gap in our understanding of how and to what extent these tools are being used, and raises important questions about the factors hindering the consistent use of pain assessment tools in equine veterinary medicine.

Using semi‐structured interviews and thematic analysis, this study aimed to gain a richer understanding of current equine pain assessment practices and barriers encountered through the lens of veterinary professionals' experiences within a UK equine hospital setting. The findings may inform large‐scale investigations and the development of targeted educational initiatives.

## MATERIALS AND METHODS

### Ethics statement

Ethical approval for this study was granted by the Royal (Dick) School of Veterinary Studies (R(D)SVS) Human Ethical Review Committee (2022‐189).

### Research design

This exploratory study used an inductive qualitative approach to investigate staff attitudes at a large, single‐centre teaching equine hospital in the UK: The R(D)SVS Equine Hospital. Inductive analysis was chosen to allow themes to be identified organically from participants’ responses rather than fitting data into pre‐existing frameworks, enabling a richer understanding of their experiences.^23^ To ensure transparency, comprehensive reporting adhered to the Consolidated Criteria for Reporting Qualitative Research (COREQ) checklist. At the time of data collection, pain scoring at the R(D)SVS Equine Hospital was routinely performed in the Intensive Care Unit (ICU) using a four‐point Numeric Rating Scale (NRS), developed in‐house and included in the standard ICU documentation. In other clinical areas of the hospital, there was no formalised or standardised pain scoring protocol in place; pain assessment was typically based on informal observation and clinical judgement.

### Data collection

A semi‐structured interview guide underwent two rounds of piloting and refinement before data collection. This flexible approach, utilising open‐ended questions, facilitated in‐depth exploration of participant perspectives on equine pain assessment.^24^ A purposive sampling approach was used to **contact** 20 veterinary professionals from diverse roles within the equine hospital via email invitation, with selection criteria informed by previous research that may influence attitudes towards pain, such as professional role, gender and experience level.^25^, ^26^ Invitations to ensure diverse perspectives were sent to all staff with interactive roles with horses, including veterinary surgeons, veterinary nurses, student veterinary nurses, technicians and grooms. An information sheet outlining the study's purpose, nature and participant expectations was provided to ensure informed consent.

**Interviews** were conducted in person at the hospital between April and June 2023 by A.E. (female, MSci BVMS, background as a mixed animal veterinary surgeon), a researcher known to participants and trained in interview techniques. A semi‐structured interview guide was used to steer the conversation while allowing themes to be identified and refined during the dialogue. All interviews were audio‐recorded with permission (Otter.ai).^27^ To minimise participant burden, each participant was interviewed once, and no separate debriefing notes were taken. Recruitment was concluded when successive interviews offered no substantively new codes or perspectives, indicating that further data were unlikely to add meaningfully to the analysis.

### Data analysis

The analysis was underpinned by a critical realist epistemology, recognising participants’ accounts as grounded in real‐world clinical practice while also shaped by sociocultural and institutional context.^28^ Reflexive thematic analysis was conducted in NVivo 14,^29^ following Braun and Clarke's six‐phase guide (2006). Theme development was iterative and interpretive, supported by reflexive engagement and theory‐informed reasoning. Rather than seeking coding consensus, the analysis aimed to identify patterns of shared meaning and underlying influences on participants’ interpretations.

Anonymised transcripts were formatted and checked for accuracy by A.E. and O.C., then coded by O.C. (female, BSc MSc, PhD candidate) and J.D. (female, BVMS SFHEA MSc DipECVAA PhD, clinician and lecturer in veterinary anaesthesia with a background in pain and ethics). Both analysts received training in reflexive thematic analysis, including guidance on coding practices and philosophical underpinnings. Coding was initially undertaken independently, then discussed to explore interpretative differences and strengthen the analysis through critical reflection. The aim was to enhance analytical depth rather than achieve consensus. Both analysts completed written reflexivity exercises to examine how their disciplinary backgrounds and assumptions might have influenced the interpretation. No transcripts were returned to participants. Full methodological details are provided in the Supporting Information.

## RESULTS

Eleven of the 20 individuals invited did not respond to the initial email invitation or declined participation due to scheduling conflicts or time constraints. In total, nine staff members took part in the study, representing a range of roles within the equine hospital. Participant roles, demographics and details are outlined in Table 1. The interviews lasted an average of 11 minutes 7 seconds ± 2 minutes 16 seconds, with durations ranging from 6 minutes 43 seconds to 14 minutes 2 seconds.

**TABLE 1:** Participant information

Identification	Gender identity	Job role	Specialism	Years in role
Student Nurse‐011	Female	Student nurse^a^	Not applicable	0–5
Technician‐012	Female	Veterinary technician	Not applicable	10–20
Nurse‐013	Female	Nurse	Not applicable	10–20
Nurse‐014	Female	Nurse	Not applicable	10–20
Vet‐021	Female	Vet	Medicine	5–10
Vet‐022	Female	Vet	Medicine	5–10
Vet‐023	Male	Vet	Surgery	5–10
Vet‐024	Male	Vet	Medicine	10–20
Vet‐025	Female	Vet	Ambulatory	20+

^a^ Student Nurse (011) previously worked as a groom for over 10 years before becoming a student nurse.

### Themes

Three main themes and nine sub‐themes were developed through the analytic process. Table 2 presents a summary of the relationship between these themes, sub‐themes and the codes from which they were constructed.

**TABLE 2:** Major themes, sub‐themes and codes

Major themes	Sub‐themes	Codes
**Assessment factors**	Challenges with existing methods	Limitations within clinical setup
		Methodological limitations
	Importance and implementation	Importance of pain assessment
		Frequency of use
	Optimising pain assessment	Optimum approach
		Positive thoughts and desires
**Observer factors**	Competence and confidence	Confidence in abilities
		Influence from education or training
		Experience gained in practice
		Experience with horses
	Subjectivity and bias	Own experience of pain
		Anthropomorphism
	Inter‐rater reliability	Role responsibilities
		Scorer variation
**Horse factors**	Individual variation	Importance of knowing the horse
		Breed or type
	Internal and external influences	Behavioural change from environmental stressors
		Diagnosis and treatment plan
		Behavioural ecology of the horse
	Observational cues	Pain‐related behaviour/facial expression
		Recognition of subtle behaviours

### Assessment factors

#### Challenges with existing methods

Participants raised concerns about the current pain assessment tools, particularly the ICU's four‐point NRS, which was described as ‘*very crude*’ (Vet‐021) and ‘*quite vague’*, the nurse added that there was *‘no indication of what we're looking at…’* (Nurse‐013).

Several felt the NRS was underused or only applied in severe cases: ‘*It's not used enough’* (Vet‐022); *‘only when we think the horse is going to be very painful’* (Vet‐013). Others echoed, *‘It could definitely be used a lot better’* (Student Nurse‐011). Concerns about the tool's sensitivity were also noted, with participants explaining that subtle variations in pain may be overlooked *‘There are quite big changes within that grade: one or zero’* (Vet‐022).

Composite scales were considered more detailed but rarely implemented. One participant reflected on their limited feasibility in a clinical setting, describing them as ‘*time‐consuming’*, and adding that *‘students feel a bit overwhelmed’* and *‘if that takes an extra 10 minutes, people don't want to spend that time’* (Vet‐021).

Pain assessment was frequently described as subjective: *‘It's pretty much all subjective’* (Vet‐022), and difficult to sustain across a rotating team: *‘If you've got the chop and change of who is doing it, it's a bit more difficult’* (Nurse‐013).

#### Importance and implementation

Participants generally endorsed the value of structured pain assessment, but described its application as inconsistent in practice. Pain scoring was described as ‘*underused*’ (Vet‐022), ‘*not routine’*, although they were *‘encouraged to use pain scales in research studies’* and found facial grimace scale papers *‘quite helpful’* but *‘don't use them quantitatively’*, instead applying elements *‘as more of a qualitative subjective assessment’* (Vet‐024). Another said the team *‘sometimes attempt[ed] to use the grimace scoring’* in the hospital, but felt it was *‘not particularly well described in horses’* (Vet‐022), while a third commented that such tools remain *‘in their infancy’* despite some promising publications (Vet‐021).

Others reported relying on informal methods. One technician explained they relied on ‘*judgement and… previous experience’* rather than scoring (Technician‐012), while a vet added they were ‘*embarrassed to say I couldn't [list] them*… *we just use our judgement’* (Vet‐025).

Some said they ‘*don't do [pain scoring] very often’* (Vet‐023), or used it only descriptively for conditions like colic or laminitis, noting it was *‘not actually used [d] day to day’* (Vet‐024). A nurse commented that pain scoring *‘could definitely be used a lot better’* (Student Nurse‐011), and another recalled efforts in a previous role: *‘We changed a lot… making sure we do it twice a day’* (Nurse‐013).

One vet commented that pain assessment is ‘not paid enough attention to’ and described it as ‘not used enough’ (Vet‐022). Another summed it up: ‘In an ideal world… we would pain score every horse with a painful condition. The reality is no, that doesn't happen’ (Vet‐021).

#### Optimising pain assessment

Participants who were familiar with pain assessment, expressed a general optimism about improving pain assessment and a growing awareness of its importance. Recognition and assessment of pain was described as *‘a really interesting topic’* (Vet‐022) and *‘I think it's really important’* (Student Nurse‐011). When it came to pain scoring, one nurse described themselves as ‘a big believer in [it]’ (Nurse‐013), while others called it ‘a useful tool’ (Vet‐021) and noted, ‘in a hospital environment I think it's definitely handy’.

Several participants also spoke about ‘growing awareness’, including ‘more of a social requirement to consider [pain]’ (Vet‐025), and a belief that scoring ‘should be used with every horse’ to detect meaningful change (Student Nurse‐011).

Although not always the case in practice, pain assessment was described as something that should involve the whole team. ‘It needs to be everyone’, one nurse explained (Nurse‐014), while a vet added, ‘I don't think you can narrow it down to one person… it will always be a team approach’ (Vet‐025).

### Observer factors

#### Competence and confidence

Participants frequently reflected on their ability to recognise pain, particularly when signs were subtle or overlapped with other behavioural presentations. One vet reflected, *‘I don't think my ability, or our ability as vets, is particularly good at diagnosing low‐grade pain’* (Vet‐022). Another noted the difficulty of *‘distinguishing pain from… dullness’* (Vet‐024), while one participant admitted they had *‘probably blocked [pain] off’* at times, *‘for not knowing what to do… or not wanting to see it’* (Vet‐025).

However, many described growing confidence over time. One vet said they had learned to *‘appreciate smaller signs’* (Vet‐022), while another described becoming *‘more alert to what sort of causes would cause different reactions’* (Nurse‐014). Several linked this to increased clinical exposure: *‘Working in this environment… you see more variety’*, which made it *‘much easier to see’* pain compared to field practice (Nurse‐013). Others noted that experience helped them focus more on the horse and *‘less on what I look like’* (Vet‐025).

Education and training were mentioned as helping with recognition. One student nurse remarked, *‘Probably now, doing nurse training… [I'm] reading them a bit better’* (Student Nurse‐011), while another participant noted, *‘There's a lot [now] in vet training about recognising pain’* (Vet‐024).

#### Subjectivity and bias

Participants described how personal experiences and emotional responses could influence how pain was recognised and managed. For some, direct experience with pain increased their empathy. One participant noted, *‘There's one thing that's made me think I need my patients better… it's probably experiencing it myself’* (Vet‐025), particularly in relation to dental procedures and childbirth. They added, *‘I know how I feel… and I don't want my patients or the owners to feel like that’*.

Subjective interpretation also shaped clinical assumptions. One vet admitted they sometimes *‘assume pain must be there based on extrapolation from humans’* (Vet‐024), even when behavioural signs were absent.

Owners were described as sometimes projecting their own experiences onto the horse, for example, saying ‘that must have been painful’ based on what they themselves found unpleasant (Vet‐023). These comments were often ‘taken with a pinch of salt’ or described as ‘emotionally driven’ (Vet‐023), and some participants raised concerns about potential anthropomorphism. One vet reflected that owners may ‘bring… their own opinion into the situation’ (Vet‐022).

#### Inter‐rater reliability

Differences in scores were attributed to two mechanisms identified during the coding process. Continuity of contact (grooms seeing horses daily vs. clinicians episodically) created unequal baselines for judging change. In parallel, professional education and experiences shaped which behaviours were accepted as pain indicators. These mechanisms account for convergence among grooms and divergence between owners, students and veterinary surgeons.

Participants described variability in how pain was assessed across different observers. Experience, training and familiarity with the horse were seen as key influences on recognition and interpretation. One vet remarked, *‘I wouldn't consider every single opinion the same’* (Vet‐021), and another noted, *‘You [and] I might look at something differently’* (Vet‐024).

Grooms were widely valued for their consistency and familiarity with individual horses. One vet explained: ‘I really use our grooms on the yard… their judgement of “oh, this horse is more painful than yesterday” is often more useful than mine, who would walk out once a day’ (Vet‐022). Others echoed that those who ‘see it day in, day out… are the most important to listen to’ (Vet‐022), particularly when trained in recognising pain.

Owner's ability was described as highly variable. Some were seen as recognising changes but not linking them to pain, while others were said to *‘vastly underestimate or overestimate’* signs (Vet‐024). As one participant put it, *‘Interpretation of behaviour is variable… some owners are very good. Others ignore it or over‐interpret it’*.

### Horse factors

#### Individual variation

Participants emphasised that horses vary widely in how they express pain, and that accurate assessment depends on understanding the individual. One vet remarked that *‘they all act differently’* and have *‘a different attitude’* (Vet‐021). Several described how subtle changes were only meaningful in context: *‘It definitely boils down to understanding each horse individually… and how that's changed’* (Nurse‐014).

Assessing unfamiliar horses, particularly during short admissions, was described as especially challenging. One vet noted, *‘We don't recognise their changes in demeanour particularly well… and we don't appreciate that as pain’* (Vet‐022).

Participants also discussed variation in behavioural response. Some horses were said to *‘soldier on’* (Vet‐021) despite pain, while others reacted more overtly. One technician described horses *‘not that lame but [with] a serious injury’*, and others who reacted more strongly to *‘something not serious’* (Technician‐012).

While breed‐based labels such as *‘stoic cob’* or *‘wussy’* thoroughbred were used, a few participants questioned their reliability: *‘We group breeds quite a lot… but I don't really have any evidence to justify our decision’* (Vet‐022).

#### Internal and external influences

Participants described how environmental stressors and physiological states could interfere with accurate pain assessment. Hospital settings and transport were frequently mentioned as external factors that altered equine behaviour. One vet explained that *‘taking the animal out of its home environment… changes the way it behaves’*, adding, *‘even an owner would find it difficult’* to interpret signs in that context (Vet‐023).

Others described how horses might mask discomfort when unsettled or on high alert. One vet reflected, ‘They're not going to show pain when they're in a new environment with new people… you'd expect them to shut down’ (Vet‐023).

Physiological arousal, particularly adrenaline, was also described as suppressing pain behaviours shortly after arrival. One vet noted that horses *‘just got off the transport… they're more excited… they show less signs of pain’* (Vet‐024).

Short stays were considered especially problematic. One vet explained, *‘We don't recognise their changes in demeanour particularly well’*, and added that after changing *‘environment and meds’* simultaneously, clinicians often *‘don't really know’* what led to improvement (Vet‐022).

#### Observational cues

Participants emphasised that while overt behaviours, such as rolling, lameness or reluctance to move, were widely recognised, more subtle signs were often missed, particularly by less experienced observers. These included deviations from a horse's usual demeanour, such as being *‘just a bit quiet’* (Vet‐024) or *‘not acting normally’* (Nurse‐013), which were not always associated with pain. One participant noted, *‘It's the big things that are noticed’* (Vet‐024).

Some described relying on general impressions, such as whether a horse was *‘standing in the back of a box’* or *‘wanting to interact’* (Vet‐023), before linking these to possible pain indicators.

Facial expressions were commonly mentioned but perceived as under‐recognised. One student reflected that the horse ‘just looked sore’ (Student Nurse‐011), while another participant explained that recognising such cues is a skill ‘acquired later on’ (Vet‐021). Specific features included pinned ears, nostril flare and *‘eyes being dull’* (Student Nurse‐011; Nurse‐014). Some acknowledged the limitations of current tools, stating that *‘face grimace scales are… in their infancy’* (Vet‐021).

As one participant summed up, *‘It doesn't take lying on the floor to be sore’* (Vet‐024).

## DISCUSSION

These preliminary results suggest that participants valued pain assessment tools, reflecting similar findings in small animal practice, where 80.3% of veterinary nurses deemed pain scales clinically useful.^19^ Despite this recognition, this study found that the tools were used inconsistently at this equine hospital, with the NRS primarily confined to use in the ICU. The NRS was widely described as vague and crude. These concerns are consistent with previous critiques of unidimensional scales, which are often viewed as overly simplistic, subjective and vulnerable to observer bias.^30^, ^31^ In response to the limitations associated with unidimensional scales, validated multidimensional tools, such as the Glasgow Composite Measure Pain Scale, have been developed in small animal medicine to capture behavioural and emotional dimensions.^32^ Although several equine‐specific multidimensional tools exist,^33^, ^34^, ^35^, ^36^, ^37^, ^38^, ^39^ such as the Unesp–Botucatu Horse Acute Pain Scale,^40^ the Orthopaedic Composite Pain Scale^41^ and the Equine Utrecht University Scale for Composite Pain Assessment,^42^ no universally accepted ‘gold standard’ has emerged.^15^ None of our participants reported the routine use of composite or facial expression‐based tools. While such tools, as the Horse Grimace Scale, have gained research interest,^43^, ^44^, ^45^, ^46^ participants viewed them as poorly described and difficult to apply, reflecting broader concerns about the disconnect between tool development and clinical adoption.^47^ Interpretive ambiguity, limited guidelines and perceived impracticality in time‐pressured settings likely compound these barriers.

Interestingly, although time constraints have been identified as a barrier to using other scales, for example, quality‐of‐life assessments in veterinary medicine,^17^ only one participant explicitly mentioned this. This may reflect the interview structure, which did not prompt selection from predefined options. Instead, participants emphasised challenges related to clinical setup, vague protocols and limited training. These findings are consistent with observations in human healthcare, where pain tool uptake depends on factors such as local resources, clinical guidelines and competing priorities.^48^, ^49^ Despite recognising the value of pain tools, staff often defaulted to informal judgement, citing contextual limitations and role‐based expectations.

Veterinary surgeons, in particular, reported a lack of confidence in recognising low‐grade pain. This finding is consistent with previous research, which has shown that veterinary surgeons often feel less confident than owners in assessing pain.^10^ However, our data indicate this uncertainty extended across roles, suggesting that limitations arise not only from professional training but also from the available tools and techniques for pain assessment.

The lack of confidence in recognising pain‐related behaviours likely reflects, at least in part, insufficient knowledge, stemming from limited training and exposure.^22^, ^50^ Around 65% of veterinary surgeons report learning pain management experientially rather than through formal education,^51^, ^52^ suggesting that limited exposure to pain assessment tools during training may therefore hinder effective practice.

Participants also reported an increased awareness of behavioural expression following graduation and clinical exposure, supporting the idea that increased knowledge and experience can improve confidence in pain assessment.^8^ Some expressed optimism about improving pain assessment practice through further training and teamwork, framing it as a shared responsibility.

This view is supported by existing research, which indicates that veterinary students value pain assessment training, and veterinary surgeons who participate in continuing education programmes are more likely to use pain scales and report greater confidence in pain assessment.^53^ Insights from human medicine similarly indicate that education is associated with more positive attitudes toward pain assessment.^54^ Together, these findings highlight the importance of targeted education focused on recognising subtle behavioural signs and utilising pain assessment tools.

The findings demonstrated that staff across roles recognised the importance of considering individual variation in behavioural indicators of pain, noting similar challenges. Regardless of time spent with horses, assessing unfamiliar animals was often difficult. Factors such as breed stereotypes, temperament differences like stoicism and neuroticism,^55^ and individual characteristics such as past experiences, age and gender can also influence pain expression.^56^ External factors, including transport and the unfamiliar hospital environment, may also contribute to difficulties in assessing pain.^57^, ^58^

Interpretations of pain were shaped by both familiarity with the animal and clinical experience. Owners, closely attuned to their horses, may recognise subtle behavioural changes, but their interpretations can be influenced by anthropomorphism, potentially leading to misjudgement.^59^, ^60^ Nevertheless, cross‐species research suggests that owners can detect subtle signs of pain in their animals,^61^ a capacity which may be under‐recognised in clinical contexts. Veterinary professionals, on the other hand, often manage more severe clinical presentations; repeated exposure to overt pain behaviours may desensitise them to more nuanced behavioural changes.^62^ While staff acknowledged the importance of owners’ familiarity with their horses, they frequently expressed scepticism about owners’ ability to interpret behavioural indicators accurately. Even when recognising their own uncertainty in identifying pain, staff generally placed greater trust in their own clinical judgement, reflecting findings from previous research.^11^ Similar patterns in pain perception have been observed in human medicine, where experienced clinicians tend to perceive lower pain intensity than their less experienced counterparts,^63^ likely due to habituation to more severe pain cases.^64^

Participants described variation in how different members of the veterinary team approached pain assessment. Differences between grooms, students, nurses and veterinary surgeons were shaped by time spent with the horse, clinical experience and role expectations. Nurses and grooms, who maintained closer and more consistent contact, were viewed as more attuned to subtle behavioural changes. In contrast, veterinary surgeons were typically more focused on technical or diagnostic aspects of care. This variation mirrors human healthcare, where nurses often prioritise patient comfort while doctors focus on procedures.^65^, ^66^ While incorporating multiple perspectives may enhance pain assessment,^67^ it can also introduce inconsistency and disagreement,^68^ highlighting the need for shared assessment frameworks.

### Limitations

This study provides preliminary insights, though several constraints must be acknowledged. Data were collected at a single site, the Royal (Dick) School of Veterinary Studies Equine Hospital. Although its multidisciplinary caseload and referral‐teaching structure are broadly typical of UK equine hospitals, the single‐site design inevitably limits transferability. Consistent with sample sizes used in other focused qualitative studies,^69^, ^70^, ^71^ only nine interviews were conducted, and the mean duration was approximately 11 minutes; time‐pressured scheduling, particularly for grooms, technicians and nurses, who lack protected time for research participation, restricted probing and follow‐up questions. This may also explain the limited representation of grooms, as only one participant had prior experience in that role. While the brief interview format has precedent and validity in fast‐paced clinical contexts where participant burden must be minimised,^72^ it may have constrained depth in this setting. Future studies will address these limitations through targeted recruitment and more flexible scheduling. Although later interviews reinforced earlier themes, supporting the sufficiency of the dataset for this study's exploratory aims, the modest sample size and brevity of interviews warrant cautious interpretation.

Additionally, the interviewer (A.E.), a veterinary surgeon senior to some participants, might have introduced power imbalances or social desirability effects, and A.E.’s limited qualitative experience could have influenced depth and direction of the conversations.^73^ The analytical lens was inevitably shaped by researcher positionality: O.C.’s long‐term equine ownership and welfare focus, and J.D.’s extensive experience as an anaesthetist, researcher in pain recognition and horse owner foregrounded particular sensitivities toward analgesic decision‐making; reflexive journalling and peer‐debriefing (see Appendix S1) were therefore employed to surface and critically examine these influences. Collectively, these factors reinforce the exploratory nature of the findings and highlight the need for larger, multi‐centre studies.

## CONCLUSION

This preliminary study provides novel insight into veterinary staff attitudes toward equine pain assessment based on interviews conducted at a single UK equine teaching hospital. A complex interplay of factors, including clinical setup, limited time with horses, limited knowledge and constraints of existing tools, contributed to the infrequent use of structured pain assessment methods.

The findings suggest a disconnect between available tools and their practical implementation. This gap appears to be shaped by contextual barriers as well as professional expectations. Although exploratory and limited in scope, the study highlights how systemic factors influence clinicians’ approaches to recognising and scoring pain.

Participants expressed a strong desire for clearer guidance, enhanced tools and more consistent protocols to support pain assessment in practice. Future multi‐site research could explore these issues more broadly and evaluate how clinical environments, team roles and institutional culture interact to shape pain recognition. Incorporating behaviour change frameworks may also support the development of effective strategies to improve uptake and, ultimately, improve equine welfare.

## AUTHOR CONTRIBUTIONS

Alice Everett, Juliet Duncan and Olivia Curry conceptualised the study. Alice Everett collected the data. Olivia Curry and Juliet Duncan performed the data analysis and interpreted the results. Olivia Curry wrote the manuscript. Olivia Curry, Juliet Duncan, Gemma Pearson and Cathy Dwyer reviewed the manuscript.

## CONFLICT OF INTEREST STATEMENT

The authors declare no conflicts of interest.

## FUNDING INFORMATION

This work was supported by the UKRI Biotechnology and Biological Sciences Research Council (BBSRC) grant number BB/T00875X/1. For the purpose of open access, the author has applied a Creative Commons Attribution (CC BY) licence to any Author Accepted Manuscript version arising from this submission.

## ETHICS STATEMENT

Ethical approval for this study was granted by the Royal (Dick) School of Veterinary Studies (R(D)SVS) Human Ethical Review Committee (2022‐189).

## Supporting information



Supporting Information

Supporting Information

## Data Availability

The data supporting the findings of this study consist of qualitative interview responses from veterinary staff within the authors' professional network. Due to privacy and ethical restrictions, interview transcripts cannot be made publicly available as they contain potentially identifiable information, even in an anonymised format. Written consent was obtained from all participants for the use of direct quotes in publications. As a result, only the interview guidelines are available as supplementary material with this article. A summary of quotes supporting the thematic analysis can be requested from the Human Ethical Review Committee at herc.vets@ed.ac.uk.
