# Supplementary material for: Preliminary investigation of equine veterinary hospital staff attitudes towards pain assessment in a single centre
Source: Vet Rec. 2025 Dec 4;198(12):e550–9. doi: 10.1002/vetr.6122 (PMC13261779; doi:10.1002/vetr.6122)
Supplement: Supplementary file 1 — Supporting Information [file VETR-198--s001.docx]

## Supplementary Methodological Details

### Epistemological stance

The analysis was underpinned by a **critical-realist** position, acknowledging that participants’ narratives reflect genuine clinical phenomena yet are mediated by sociocultural context. This stance guided coding decisions and thematic interpretation.

### Conceptual orientation and quality considerations in reflexive TA

- Reflexive thematic analysis (RTA) treats **researcher subjectivity as an analytic resource** rather than a bias to eliminate.
- Themes are **constructed as patterns of shared meaning** underpinned by a central organising concept— not merely topic summaries.
- The analytic process is **recursive and iterative;** coding and theme development are revisited as understanding deepens.
- This study employs Braun & Clarke’s **reflexive** variant exclusively, distinct from coding-reliability or codebook TA (Braun, Clarke & Weate 2019).
- Coding-reliability metrics were not calculated; instead, quality was assured through reflexive memos, peer debriefing and a transparent audit trail (see below).
- Updated terminology (e.g. identifying or generating themes) is used throughout the manuscript.

## Reflexive Thematic Analysis: Summary of the Six Phases

*(Adapted from Braun & Clarke, 2006; informed by Wiltshire & Ronkainen, 2021; Naeem et al., 2023)*

| **Phase** | **Activities** | **Reflexive and Interpretive Considerations** |
| --- | --- | --- |
| **1. Familiarisation** | Transcripts were read several times and uploaded into NVivo 14. Early notes and observations were recorded. | Researchers documented their first impressions and any assumptions. Moments of uncertainty or contradiction in the data were flagged for further attention. |
| **2. Generating initial codes** | OC and JD coded the transcripts independently, using both surface-level and deeper, interpretive codes. | Coding was treated as interpretive rather than objective. Researchers discussed differences in perspective and reflected on how their backgrounds shaped what they noticed. |
| **3. Constructing themes** | Coded segments were grouped into early themes and subthemes based on repeated ideas or meanings. | Themes were shaped through back-and-forth thinking between what participants said and what it might mean. Researchers aimed to build meaningful patterns, not just summaries. |
| **4. Reviewing themes** | Themes were refined by checking them against the original data and adjusting subthemes as needed. | Decisions were made based on clarity, relevance, and how well the themes explained the data. Researchers also considered what broader factors (e.g. workplace culture) might shape participant views. |
| **5. Defining and naming themes** | Each theme was clearly described and named. A final map showing theme relationships was created. | Theme names were chosen to reflect both what participants said and the deeper ideas behind them, avoiding labels that were too general or obvious. |
| **6. Producing the report** | A narrative summary of each theme was written and supported with selected participant quotes. Themes were discussed in relation to existing literature and the study’s aims. | Researchers reflected on how their interpretations were shaped by their positions and knowledge. The goal was to offer useful insights, not claim absolute truth. |

**Reflexivity statements (abridged)**

**OC (primary analyst – PhD candidate, BSc MSc)**

- Long-standing horse owner; prior experience managing pain in own horses heightened sensitivity to behavioural subtleties.
- Personal surgery experience and welfare orientation motivated an empathetic reading of the data.
- Reflexive journalling used to bracket these assumptions.

**JD (secondary analyst – clinician-lecturer, BVMS SFHEA MSc DipECVAA PhD)**

- Experienced equine anaesthetist, pain-management researcher and horse owner; pragmatic clinical lens foregrounded analgesic decision-making.
- Maintained reflexive notes to monitor potential bias arising from clinical authority and motherhood responsibilities.
