# Supplementary material for: Preliminary investigation of equine veterinary hospital staff attitudes towards pain assessment in a single centre
Source: Vet Rec. 2025 Dec 4;198(12):e550–9. doi: 10.1002/vetr.6122 (PMC13261779; doi:10.1002/vetr.6122)
Supplement: Supplementary file 2 — Supporting Information [file VETR-198--s002.pdf]

Equine Pain interviews Spring 2023

Name: **Date:** **Time:** **Location:**

<u>Interview schedule</u>

1. Go through confidentiality
2. Explain reason for interview
3. Topics & questions

Topics/ questions	Question prompts (if respondents need encouragement)
Q. Can you tell me about your experience in the veterinary profession/working with horses?	1. How long have you been qualified for?<br>2. Have you worked in general practice?<br>3. What species have you worked with?
Q. What is your experience of pain yourself/ in other people?	1. Have you previously experienced pain? Describe….<br>2. Have you seen another person in pain?<br>3. What is your attitude towards pain in other people?<br>4. What is your experience of pain relief in people?
Q. Are you able to tell when a horse is experiencing pain?	1. Who do you think can assess pain?<br>2. Who taught you how to assess pain?<br>3. Has your judgement of pain changed with time?
Q Do you know of any tools which are available to help assess pain?	1. Do you use (mentioned tools)<br>2. If so, in what situation?<br>3. Do you feel it changes the way you respond compared to simple observation? How?<br>4. Do you ask for a second opinion?<br>5. How do you respond to pain (ie a pain score over threshold) in your role? /What do you do when you see a horse is painful?

Equine Pain interviews Spring 2023

Q. Why do you think horses show pain?	
Q. Who would be best at assessing Pain in horses? In a Hospital setting? At home?<br>Q. Do you have any thoughts or comments on this interview?	1. Are owners equipped to assess pain as well as veterinary professionals?
